# Prevalence of Seizures in Hospitalizations with Traumatic Brain Injury: A U.S. Population-Based Study

**DOI:** 10.1089/neur.2025.0001

**Published:** 2025-04-09

**Authors:** Alka Mithal, Maanek Sehgal, Christopher Newey, Derek Ems, Vince Florio, Gurkirpal Singh

**Affiliations:** ^1^ Institute of Clinical Outcomes Research and Education (ICORE), Woodside, California, USA.; ^2^ Sanford USD Medical Center, Sioux Falls, South Dakota, USA.; ^3^ UCB, Smyrna, Georgia, USA.

**Keywords:** costs, database analysis, epilepsy, length of stay, national inpatient sample, traumatic brain injury

## Abstract

In the United States, data on outcomes in adults hospitalized with traumatic brain injury (TBI) and concomitant seizures are limited. Here, we report on a feasibility analysis to understand the prevalence and consequences of concomitant seizures in patients with TBI. A retrospective database study was conducted using the National Inpatient Sample 2016–2020. Hospitalizations in patients (≥18 years of age) with TBI were assessed and stratified into groups either with or without concomitant seizures. All patient data were stratified by age, sex, ethnicity, and payer type. The primary outcome was the prevalence of seizures or epilepsy among hospitalizations for TBI. Other outcome variables recorded were mean charges, length of hospital stay, and case fatality. Overall, 1,591,575 hospitalizations with TBI were assessed over the study period. TBI prevalence remained relatively constant throughout the study period and was higher in men and those aged ≥65 years. Concomitant seizures were observed in 12.2% of all patients and were highest for men, the 45–64 years age group, and Black and Native Americans. Mean charges were significantly higher and length of hospital stay was significantly longer in TBI hospitalizations with seizures compared with those without seizures across all study years. No significant difference in case fatality between patients with seizures compared with those without seizures was observed. Data from this analysis showed differences in demographics and outcomes for TBI hospitalizations with versus without concomitant seizures, highlighting potential disparities in health care for patients experiencing seizures that warrant further research.

## Introduction 

In the United States in 2013, approximately 2.8 million people experienced a traumatic brain injury (TBI).^1^ TBI is associated with a higher risk of seizures (which includes epilepsy), affecting 2.7% of adults^2^ and 10% of children,^3^ following a TBI.

Although it is known that TBI is associated with a higher risk of seizures post-injury, little is known about how the development of seizures in patients with TBI affects outcomes.^4–9^ Studies have reported increased length of stay for adults hospitalized with TBI and concomitant seizures (early post-traumatic seizures [PTS]^10^ and post-traumatic epilepsy [PTE]^11^), as well as higher in-hospital mortality for those with PTE,^11^ compared with those hospitalized with TBI without concomitant seizures.

Limited data are available on the outcomes of U.S. adults with TBI and concomitant seizures. To address this paucity of data, a feasibility analysis was conducted using a national U.S. hospitalization database to understand the prevalence and consequences of concomitant seizures in adults with TBI.

## Methods

A retrospective database analysis was conducted using the National Inpatient Sample (NIS)^12^ from 2016 to 2020. The NIS is a random and stratified sample of U.S. community hospitals. At the national level, it can generate estimates of utilization, inpatient costs, and outcomes, and provide information on all patients regardless of payer (including Medicare, Medicaid, private insurance, and the uninsured). It uses unweighted data from approximately 7 million hospitalizations per year and, using weighted data, it can estimate around 35 million hospitalizations across the U.S.

To construct the dataset, the NIS was used to retrieve data on all hospitalizations in patients aged ≥18 years with a primary or secondary diagnosis of TBI using the relevant International Classification of Diseases, 10th Revision, Clinical Modification (ICD-10-CM) codes (Supplementary Table S1) between January 1, 2016, and December 31, 2020. These hospitalizations were then analyzed for a concomitant primary or secondary diagnosis of seizures or epilepsy (defined as a hospitalization having ≥1 of the relevant ICD-10-CM codes from Supplementary Table S2). All data were anonymized.

The primary outcome was the prevalence of seizures or epilepsy among hospitalizations for TBI. Other outcome variables recorded were mean hospital charges, length of hospital stay, and case fatality (hospitalizations resulting in death).

Hospitalizations with TBI were classified into two groups according to whether or not there was a recorded diagnosis of seizures or epilepsy during hospitalization. The prevalence of TBI hospitalizations with seizures was calculated as a percentage of the total hospitalized population with TBI for each calendar year. The demographic variables evaluated were described in groups with and without seizures for age, sex, race/ethnicity, payer type, charges (reported as USD for each calendar year), length of stay, and case fatality. National estimates and demographics of patients hospitalized with TBI with and without seizures, standard error of the mean, and 95% confidence limits were calculated using the Statistical Analysis Software program (version 9.4). The Welch *t*-test was used to compare differences in outcomes among patient hospitalizations with TBI with and without seizures.

## Results

There were more than 300,000 hospitalizations each year (2016–2020) with a primary or secondary diagnosis of TBI, with a total of 1,591,575 hospitalizations over the study period. The annual prevalence of TBI hospitalizations remained relatively constant at 124–128 per 100,000 of the U.S. study population from 2016 to 2020 (Table 1). Men had a higher prevalence of TBI hospitalizations than women, and the prevalence of TBI hospitalizations was highest in patients ≥65 years of age for both males and females.

**Table 1.** Prevalence of Hospitalizations for Traumatic Brain Injury in the U.S. Population, and Percentage of Seizures or Epilepsy in Traumatic Brain Injury Hospitalizations

	2016	2017	2018	2019	2020
Hospitalizations, *n* per 100,000 U.S. population					
Overall	124	126	127	128	124
Males	155	157	157	159	157
18–44 years	99	95	91	89	93
45–64 years	130	132	130	132	132
≥65 years	353	367	374	379	355
Females	95	96	97	99	92
18–44 years	36	35	34	32	33
45–64 years	56	57	58	58	55
≥65 years	278	280	281	286	253
Hospitalizations with diagnoses of interest					
TBI, *n*	309,755	316,660	320,745	327,125	317,290
Seizures or Epilepsy in those with TBI, *n* (%)	36,345 (11.7)	37,310 (11.8)	39,950 (12.5)	41,325 (12.6)	39,680 (12.5)

TBI, traumatic brain injury; U.S., United States.

Of hospitalizations with TBI, 194,610 (12.2%) had a concomitant primary or secondary diagnosis of seizures or epilepsy. There was a slight increase in the proportion of seizures or epilepsy in TBI hospitalizations in 2020 (12.5%) compared with 2016 (11.7%) (Table 1).

The total TBI hospitalizations with and without seizures by study year are summarized in Table 2. From 2016 to 2019, approximately 65% of hospitalizations with TBI and seizures were men, which increased to 66.7% in 2020. The age group with the highest percentage of seizures in TBI hospitalizations was 45–64 years for both men and women, although the percentage was slightly higher in males. Across all years in the study period, patients hospitalized for TBI with seizures had a younger mean age than those hospitalized without seizures. Among racial groups, a higher proportion of the Black and Native American populations had seizures in TBI hospitalizations versus other groups across all study years. Medicare and Medicaid paid for 70.6–71.8% of the TBI hospitalizations with seizures and 61.5–64.6% of the TBI hospitalizations without seizures across the study period.

**Table 2.** Demographics, Charges, Length of Stay, and Case Fatality in the U.S. Population Hospitalized with TBI, With and Without Seizures or Epilepsy From 2016 to 2020

Characteristic/Outcome	2016 (*N* = 309,755)		2017 (*N* = 316,660)		2018 (*N* = 320,745)		2019 (*N* = 327,125)		2020 (*N* = 317,290)	
	With seizures/epilepsy	Without seizures/epilepsy	With seizures/epilepsy	Without seizures/epilepsy	With seizures/epilepsy	Without seizures/epilepsy	With seizures/epilepsy	Without seizures/epilepsy	With seizures/epilepsy	**Without seizures/epilepsy**
Hospitalizations, *n* (%)	36,345 (11.7)	273,410 (88.3)	37,310 (11.8)	279,350 (88.2)	39,950 (12.5)	280,795 (87.5)	41,325 (12.6)	285,800 (87.4)	39,680 (12.5)	277,610 (87.5)
95% LCL—UCL	34,605–38,085	259,189–287,631	35,457–39,163	265,193–293,507	38,012–41,888	267,053–294,538	39,335–43,315	271,763–299,837	37,815–41,545	263,840–291,379
Age, mean, years	59.0	60.9	58.8	61.8	59.8	62.5	60.0	63.2	59.2	62.3
95% LCL—UCL	58.4–59.5	60.3–61.4	58.3–59.4	61.3–62.3	59.2–60.3	62.0–62.9	59.5–60.5	62.8–63.7	58.7–59.7	61.8–62.8
Sex, *n* (% of subpopulation per calendar year)										
Male	23,485 (12.5)	165,145 (87.5)	24,270 (12.6)	168,590 (87.4)	26,080 (13.4)	168,345 (86.6)	26,875 (13.6)	171,090 (86.4)	26,465 (13.5)	170,290 (86.5)
95% LCL—UCL	22,260–24,710	155,938–174,352	22,939–25,601	159,383–177,797	24,716–27,444	159,534–177,156	25,484–28,266	162,095–180,085	25,116–27,814	161,223–179,356
18–44 years	6540 (11.2)	51,670 (88.8)	6430 (11.5)	49,410 (88.5)	6630 (12.2)	47,500 (87.8)	6705 (12.7)	46,290 (87.3)	7005 (12.7)	48,370 (87.3)
95% LCL—UCL	6043–7037	47,716–55,624	5919–6941	45,735–53,085	6114–7146	44,010–50,990	6195–7215	42,917–49,663	6467–7543	44,836–51,904
45–64 years	8620 (16.1)	44,910 (83.9)	9025 (16.6)	45,290 (83.4)	9125 (17.2)	44,020 (82.8)	9395 (17.5)	44,345 (82.5)	9110 (17.1)	44,225 (82.9)
95% LCL—UCL	8059–9181	42,037–47,783	8402–9648	42,469–48,111	8516–9734	41,397–46,643	8773–10,018	41,656–47,034	8534–9686	41,545–46,905
≥65 years	8325 (10.8)	68,565 (89.2)	8815 (10.7)	73,890 (89.3)	10,325 (11.8)	76,825 (88.2)	10,775 (11.8)	80,455 (88.2)	10,350 (11.8)	77,695 (88.2)
95% LCL—UCL	7792–8858	65,392–71,738	8267–9363	70,492–77,288	9713–10,937	73,394–80,256	10,136–11,414	76,753–84,157	9734–10,966	74,131–81,259
Female	12,860 (10.6)	108,145 (89.4)	13,030 (10.5)	110,715 (89.5)	13,865 (11.0)	112,405 (89.0)	14,445 (11.2)	114,635 (88.8)	13,215 (11.0)	107,200 (89.0)
95% LCL—UCL	12,124–13,596	102,832–113,458	12,313–13,747	105,449–115,980	13,096–14,634	107,151–117,659	13,630–15,260	109,271–119,999	12,494–13,936	102,188–112,211
18–44 years	2275 (11.0)	18,465 (89.0)	2440 (12.1)	17,740 (87.9)	2475 (12.5)	17,350 (87.5)	2485 (13.3)	16,195 (86.7)	2355 (12.3)	16,860 (87.7)
95% LCL—UCL	2038–2512	16,980–19,950	2201–2679	16,387–19,093	2214–2736	16,028–18,672	2238–2732	14,949–17,441	2108–2602	15,529–18,191
45–64 years	3640 (15.1)	20,480 (84.9)	3795 (15.4)	20,805 (84.6)	4025 (16.3)	20,690 (83.7)	4155 (16.8)	20,580 (83.2)	3815 (16.4)	19,420 (83.6)
95% LCL — UCL	3344–3936	19,154–21,806	3480–4110	19,536–22,074	3693–4357	19,440–21,940	3819–4491	19,395–21,765	3501–4129	18,263–20,577
≥65 years	6945 (9.1)	69,200 (90.9)	6795 (8.6)	72,170 (91.4)	7365 (9.0)	74,365 (91.0)	7805 (9.1)	77,860 (90.9)	7045 (9.0)	70,920 (91.0)
95% LCL — UCL	6464–7426	66,070–72,330	6341–7249	68,957–75,383	6894–7836	71,083–77,647	7282–8328	74,365–81,355	6584–7506	67,772–74,068
Race/ethnicity, *n* (% of subpopulation per calendar year)										
White	24,015 (11.5)	185,575 (88.5)	24,460 (11.4)	189,500 (88.6)	26,355 (12.0)	193,040 (88.0)	27,355 (12.1)	199,150 (87.9)	25,825 (12.0)	188,890 (88.0)
95% LCL—UCL	22,780–25,250	175,315–195,834	23,201–25,719	179,385–199,615	25,034–27,676	183,111–202,969	26,017–28,693	188,950–209,350	24,552–27,098	179,037–198,743
Black	4975 (15.8)	26,495 (84.2)	5290 (16.2)	27,355 (83.8)	5535 (16.9)	27,165 (83.1)	6335 (18.8)	27,440 (81.2)	5925 (16.7)	29,660 (83.3)
95% LCL—UCL	4451–5499	23,714–29,276	4752–5828	24,713–29,997	4978–6092	24,496–29,834	5733–6937	24,833–30,047	5350–6500	26,665–32,655
Hispanic	3190 (10.3)	27,835 (89.7)	3640 (10.9)	29,885 (89.1)	3975 (11.3)	31,185 (88.7)	3985 (12.0)	29,355 (88.0)	4180 (12.4)	29,505 (87.6)
95% LCL—UCL	2808–3572	24,641–31,029	3240–4040	26,501–33,269	3484–4466	27,363–35,007	3531–4439	26,334–32,376	3711–4649	26,323–32,687
Asian/Pacific Islander	785 (8.7)	8260 (91.3)	865 (8.8)	8990 (91.2)	1165 (11.3)	9150 (88.7)	980 (9.4)	9485 (90.6)	850 (9.0)	8495 (91.0)
95% LCL—UCL	633–937	7116–9404	700–1030	7702–10,278	981–1349	7920–10,380	806–1154	8158–10,812	674–1026	7300–9690
Native American	460 (18.7)	1995 (81.3)	425 (18.6)	1860 (81.4)	475 (18.4)	2100 (81.6)	470 (17.4)	2235 (82.6)	530 (17.5)	2500 (82.5)
95% LCL—UCL	289–631	1429–2561	295–555	1367–2353	316–634	1495–2705	307–633	1502–2968	355–705	1768–3232
Other	1000 (10.9)	8205 (89.1)	1170 (11.4)	9110 (88.6)	1220 (11.5)	9385 (88.5)	1180 (10.8)	9715 (89.2)	1205 (11.2)	9550 (88.8)
95% LCL—UCL	805–1195	6502–9908	957–1383	7542–10,678	1012–1428	8147–10,623	979–1381	8488–10,942	991–1419	8343–10,757
Insurance payer, *n*										
Medicare	18,575	131,070	18,845	138,640	20,625	142,770	21,555	148,775	20,040	139,060
95% LCL—UCL	17,622–19,528	125,309–136,831	17,858–19,832	132,627–144,653	19,588–21,662	136,634–148,906	20,463–22,647	142,162–155,388	19,052–21,028	133,014–145,106
Medicaid	7225	37,045	7575	37,005	7580	35,945	8135	35,460	8250	40,185
95% LCL—UCL	6671–7779	33,865–40,225	7003–8147	33,965–40,045	6989–8171	33,106–38,784	7555–8715	32,635–38,285	7618–8882	37,098–43,272
Private Insurance	6735	70,900	7225	70,265	7755	67,950	7460	67,655	7410	64,805
95% LCL—UCL	6227–7243	66,103–75,697	6692–7758	65,718–74,812	7166–8344	63,488–72,412	6896–8024	63,256–72,054	6886–7934	60,443–69,167
Self-pay	1900	17,350	1905	16,600	2035	17,470	2170	16,760	1865	17,280
95% LCL—UCL	1644–2156	15,463–19,237	1639–2171	14,775–18,425	1777–2293	15,668–19,272	1887–2453	15,099–18,421	1641–2089	15,480–19,080
No charge	140	1175	175	1155	165	1365	155	1320	175	1210
95% LCL—UCL	82–198	812–1538	100–250	762–1548	100–230	969–1761	91–219	910–1730	108–242	889–1531
Other	1725	15,270	1450	14,700	1745	14,670	1795	15,275	1880	14,420
95% LCL—UCL	1473–1977	13,603–16,937	1247–1653	13,304–16,096	1518–1972	13,395–15,945	1553–2037	13,919–16,631	1642–2118	13,099–15,741
Outcomes										
Charges, mean (SEM), U.S. $	102,664 (2592)^**^	93,686 (2254)	105,959 (2775)^*^	97,178 (2171)	112,319 (2699)^**^	101,965 (2366)	119,373 (3154)^**^	108,164 (2513)	132,017 (3651) *	120,816 (2835)
95% LCL—UCL	97,582–107,747	89,266–98,105	100,518–111,399	92,921–101,435	107,027–117,611	97,326–106,603	113,190–125,556	103,237–113,090	124,860–139,175	115,259–126,374
Length of stay, mean (SEM), days	9.0 (0.2)^***^	7.0 (0.1)	8.6 (0.2)^***^	6.9 (0.1)	8.8 (0.2)^***^	6.9 (0.1)	8.8 (0.2)^***^	7.0 (0.1)	9.2 (0.2)^***^	7.4 (0.1)
95% LCL—UCL	8.6–9.3	6.9–7.2	8.3–9.0	6.8–7.1	8.5–9.1	6.8–7.1	8.4–9.1	6.9–7.2	8.9–9.6	7.2–7.5
Case fatality, mean (SEM), %	6.9 (0.3)	6.8 (0.1)	7.1 (0.3)	6.8 (0.1)	7.2 (0.3)	6.9 (0.1)	6.5 (0.3)	6.9 (0.1)	7.3 (0.3)	7.3 (0.2)
95% LCL—UCL	6.3–7.5	6.6–7.1	6.5–7.7	6.6–7.1	6.6–7.7	6.7–7.2	6.0–7.1	6.6–7.2	6.7–7.9	7.0–7.6

^*^ *p* < 0.05.

^**^ *p* < 0.01.

^***^ *p* < 0.0001.

LCL, lower confidence limit; SEM, standard error of the mean; UCL, upper confidence limit; U.S., United States.

Patient outcome data are also shown in Table 2. Mean charges were significantly higher in hospitalizations with TBI with seizures compared with those without seizures across all years in the study period (*p* < 0.05). Similarly, the length of hospital stay was significantly longer in hospitalizations with TBI with seizures compared with those without seizures (*p* < 0.0001). However, no statistically significant differences were observed in case fatality between TBI hospitalizations with versus without seizures across all years. Case fatality showed a small general increase between 2016 and 2020, peaking in 2020.

## Discussion

This feasibility analysis on data derived from a national U.S. hospitalization database aimed to understand the prevalence and consequences of concomitant seizures in hospitalizations with TBI. Over 1.5 million hospitalizations were analyzed over the study period (2016–2020).

We found a small increase in the proportion of seizures in TBI hospitalizations from 2016 (11.7%) to 2020 (12.5%). This is somewhat higher than that reported for early PTS^10^ and for PTE in studies that included hospitalized^13^ and non-hospitalized patients.^2,4^ However, it should be noted that this study examined all seizures and not just those following a TBI, so it is possible that some patients had pre-existing seizures, which may explain the higher proportions observed. The higher proportion may reflect the increased use of electroencephalograms to monitor brain-injured patients. It may also reflect that injury type has altered over the study period, that patients being hospitalized are more ill, or that the TBI severity level shifted over the study period, with more severe TBI requiring hospitalization occurring in 2020 compared with previous years, although the reasons for a shift such as this remain unclear. The findings in our analysis align with a study that identified risk factors for developing early PTS in patients with moderate to severe TBI,^10^ in which patients with PTS versus those without had an increased length of hospital stay and similar in-hospital mortality. This study also demonstrated poorer 2-year outcomes (i.e., development of PTE or death) versus those who did not develop PTS.^10^

In line with past studies,^1,14^ the prevalence of hospitalizations with TBI was higher in males and in older patients (≥65 years); the latter is usually attributed to the higher occurrence of falls in this age group. Overall, in our study, approximately 12% of hospitalizations with TBI had concomitant seizures. Interestingly, among racial groups, a higher proportion of the Black and Native American populations had seizures in TBI hospitalizations versus other groups across all study years. Previous evidence suggests that racial/ethnic differences exist in TBI incidence and treatment.^15^ It would be of interest to explore racial/ethnic differences in outcomes between patients with TBI with and without seizures in a future study, as racial/ethnic disparities in care have previously been noted in TBI rehabilitation outcomes.^16^

The outcome measures of the length of hospital stay and mean charges were both significantly higher in TBI hospitalizations with seizures compared with TBI hospitalizations without seizures, which may indicate that TBI resulting in seizures are more complex or severe cases. Although no significant difference in case fatality was noted in this study between TBI hospitalizations with and without seizures, future work in this area may benefit from the consideration of longer-term outcome measures, for example, mortality at 1–5 years post-TBI.

Some limitations of the current work are that, as a feasibility study, the data analyzed were limited in scope and provide association rather than causation. It is possible that some patients hospitalized with TBI had a prior epilepsy diagnosis, rather than seizures or epilepsy resulting from the TBI, and it was not possible to differentiate between these two groups in the current analysis. This study counted hospitalizations and, as data were anonymized, a patient could be counted more than once if subsequentially readmitted to the hospital. The data were based on hospitalization records and did not include information on seizure prophylactic use, timing or duration of seizures, or TBI severity or type. Furthermore, as the data involved observations made during hospitalization, patients may have developed seizures or epilepsy post-discharge and so would not be reported here.

## Conclusion

In conclusion, this feasibility analysis demonstrated the utility of the NIS database for providing robust data on seizure prevalence in hospitalizations with TBI. Here we have observed that hospitalizations with TBI with concomitant seizures experience longer stays in the hospital and higher charges compared with those without seizures. As expected, TBI prevalence was higher in males and older adults. However, an interesting finding of this study was that a higher proportion of the Black and Native American populations had seizures in TBI hospitalizations versus other groups, possibly suggestive of racial disparities in health care that warrant further investigation. Points of interest to cover in future analyses could include a more detailed analysis of seizure/epilepsy characteristics, further stratification of TBI severity and type, the inclusion of a control group, and antiseizure medication prescription at discharge.

## Data Availability

The data from non-clinical studies are outside of UCB’s data sharing policy and are unavailable for sharing.

## Transparency, Rigor, and Reproducibility Summary

This study was not formally registered because it was conducted as part of a feasibility analysis. The analysis plan was not formally pre-registered. National estimates and demographics of hospitalizations with TBI with and without epilepsy, standard errors, and 95% confidence limits were calculated using the Statistical Analysis Software program. The Welch *t*-test was used to compare differences in hospitalizations in TBI with epilepsy and in TBI without epilepsy. Overall, 1,591,575 hospitalizations with TBI were assessed over the study period. All data were de-identified. Data were collected using the National Inpatient Sample database to cover the period 2016–2020. Equipment and software used to perform acquisition and analysis including the National Inpatient Sample database are available from the Health Care Costs and Utilization Project (Rockville, MD) and SAS (Cary, NC). The key inclusion criteria are established standards in the field. Confidence intervals have been reported in the main table for all outcomes. No replication or external validation studies have been performed or are planned/ongoing at this time to our knowledge. Data from non-clinical studies are outside of UCB’s data sharing policy and are unavailable for sharing. There is no analytic code associated with this study. The authors agree or have agreed to publish the article using the Mary Ann Liebert Inc. “Open Access” option under the appropriate license.

## Abbreviations Used

ICD-10-CM: International Classification of Diseases, Tenth Revision, Clinical Modification
LCL: lower confidence limit
NIS: National Inpatient Sample
PTE: post-traumatic epilepsy
PTS: post-traumatic seizures
SEM: standard error of the mean
TBI: traumatic brain injury
UCL: upper confidence limit
US: United States
USD: United States dollar
