# Supplementary material for: Prevalence of Seizures in Hospitalizations with Traumatic Brain Injury: A U.S. Population-Based Study
Source: Neurotrauma Rep. 2025 Apr 9;6(1):291–7. doi: 10.1089/neur.2025.0001 (PMC12040531; doi:10.1089/neur.2025.0001)
Supplement: Supplementary Table S1 [file neur.2025.0001_supplementary_tables1.pdf]

Table S1. ICD-10-CM Codes Used for Diagnosis of TBI

'R561'	'S06303A'	'S06336D'	'S06369S'	'S065X3A'	'S06826D'
'S060X0A'	'S06303D'	'S06336S'	'S06370A'	'S065X3D'	'S06826S'
'S060X0D'	'S06303S'	'S06337A'	'S06370D'	'S065X3S'	'S06827A'
'S060X0S'	'S06304A'	'S06337D'	'S06370S'	'S065X4A'	'S06827D'
'S060X1A'	'S06304D'	'S06337S'	'S06371A'	'S065X4D'	'S06827S'
'S060X1D'	'S06304S'	'S06338A'	'S06371D'	'S065X4S'	'S06828A'
'S060X1S'	'S06305A'	'S06338D'	'S06371S'	'S065X5A'	'S06828D'
'S060X2A'	'S06305D'	'S06338S'	'S06372A'	'S065X5D'	'S06828S'
'S060X2D'	'S06305S'	'S06339A'	'S06372D'	'S065X5S'	'S06829A'
'S060X2S'	'S06306A'	'S06339D'	'S06372S'	'S065X6A'	'S06829D'
'S060X3A'	'S06306D'	'S06339S'	'S06373A'	'S065X6D'	'S06829S'
'S060X3D'	'S06306S'	'S06340A'	'S06373D'	'S065X6S'	'S06890A'
'S060X3S'	'S06307A'	'S06340D'	'S06373S'	'S065X7A'	'S06890D'
'S060X4A'	'S06307D'	'S06340S'	'S06374A'	'S065X7D'	'S06890S'
'S060X4D'	'S06307S'	'S06341A'	'S06374D'	'S065X7S'	'S06891A'
'S060X4S'	'S06308A'	'S06341D'	'S06374S'	'S065X8A'	'S06891D'
'S060X5A'	'S06308D'	'S06341S'	'S06375A'	'S065X8D'	'S06891S'
'S060X5D'	'S06308S'	'S06342A'	'S06375D'	'S065X8S'	'S06892A'
'S060X5S'	'S06309A'	'S06342D'	'S06375S'	'S065X9A'	'S06892D'
'S060X6A'	'S06309D'	'S06342S'	'S06376A'	'S065X9D'	'S06892S'
'S060X6D'	'S06309S'	'S06343A'	'S06376D'	'S065X9S'	'S06893A'
'S060X6S'	'S06310A'	'S06343D'	'S06376S'	'S066X0A'	'S06893D'
'S060X7A'	'S06310D'	'S06343S'	'S06377A'	'S066X0D'	'S06893S'
'S060X7D'	'S06310S'	'S06344A'	'S06377D'	'S066X0S'	'S06894A'
'S060X7S'	'S06311A'	'S06344D'	'S06377S'	'S066X1A'	'S06894D'
'S060X8A'	'S06311D'	'S06344S'	'S06378A'	'S066X1D'	'S06894S'
'S060X8D'	'S06311S'	'S06345A'	'S06378D'	'S066X1S'	'S06895A'
'S060X8S'	'S06312A'	'S06345D'	'S06378S'	'S066X2A'	'S06895D'
'S060X9A'	'S06312D'	'S06345S'	'S06379A'	'S066X2D'	'S06895S'
'S060X9D'	'S06312S'	'S06346A'	'S06379D'	'S066X2S'	'S06896A'
'S060X9S'	'S06313A'	'S06346D'	'S06379S'	'S066X3A'	'S06896D'
'S061X0A'	'S06313D'	'S06346S'	'S06380A'	'S066X3D'	'S06896S'
'S061X0D'	'S06313S'	'S06347A'	'S06380D'	'S066X3S'	'S06897A'
'S061X0S'	'S06314A'	'S06347D'	'S06380S'	'S066X4A'	'S06897D'
'S061X1A'	'S06314D'	'S06347S'	'S06381A'	'S066X4D'	'S06897S'
'S061X1D'	'S06314S'	'S06348A'	'S06381D'	'S066X4S'	'S06898A'
'S061X1S'	'S06315A'	'S06348D'	'S06381S'	'S066X5A'	'S06898D'
'S061X2A'	'S06315D'	'S06348S'	'S06382A'	'S066X5D'	'S06898S'
'S061X2D'	'S06315S'	'S06349A'	'S06382D'	'S066X5S'	'S06899A'
'S061X2S'	'S06316A'	'S06349D'	'S06382S'	'S066X6A'	'S06899D'
'S061X3A'	'S06316D'	'S06349S'	'S06383A'	'S066X6D'	'S06899S'
'S061X3D'	'S06316S'	'S06350A'	'S06383D'	'S066X6S'	'S069X0A'
'S061X3S'	'S06317A'	'S06350D'	'S06383S'	'S066X7A'	'S069X0D'
'S061X4A'	'S06317D'	'S06350S'	'S06384A'	'S066X7D'	'S069X0S'
'S061X4D'	'S06317S'	'S06351A'	'S06384D'	'S066X7S'	'S069X1A'
'S061X4S'	'S06318A'	'S06351D'	'S06384S'	'S066X8A'	'S069X1D'
'S061X5A'	'S06318D'	'S06351S'	'S06385A'	'S066X8D'	'S069X1S'
'S061X5D'	'S06318S'	'S06352A'	'S06385D'	'S066X8S'	'S069X2A'
'S061X5S'	'S06319A'	'S06352D'	'S06385S'	'S066X9A'	'S069X2D'
'S061X6A'	'S06319D'	'S06352S'	'S06386A'	'S066X9D'	'S069X2S'
'S061X6D'	'S06319S'	'S06353A'	'S06386D'	'S066X9S'	'S069X3A'
'S061X6S'	'S06320A'	'S06353D'	'S06386S'	'S06810A'	'S069X3D'
'S061X7A'	'S06320D'	'S06353S'	'S06387A'	'S06810D'	'S069X3S'
'S061X7D'	'S06320S'	'S06354A'	'S06387D'	'S06810S'	'S069X4A'
'S061X7S'	'S06321A'	'S06354D'	'S06387S'	'S06811A'	'S069X4D'
'S061X8A'	'S06321D'	'S06354S'	'S06388A'	'S06811D'	'S069X4S'
'S061X8D'	'S06321S'	'S06355A'	'S06388D'	'S06811S'	'S069X5A'
'S061X8S'	'S06322A'	'S06355D'	'S06388S'	'S06812A'	'S069X5D'

'S061X9A'	'S06322D'	'S06355S'	'S06389A'	'S06812D'	'S069X5S'
'S061X9D'	'S06322S'	'S06356A'	'S06389D'	'S06812S'	'S069X6A'
'S061X9S'	'S06323A'	'S06356D'	'S06389S'	'S06813A'	'S069X6D'
'S062X0A'	'S06323D'	'S06356S'	'S064X0A'	'S06813D'	'S069X6S'
'S062X0D'	'S06323S'	'S06357A'	'S064X0D'	'S06813S'	'S069X7A'
'S062X0S'	'S06324A'	'S06357D'	'S064X0S'	'S06814A'	'S069X7D'
'S062X1A'	'S06324D'	'S06357S'	'S064X1A'	'S06814D'	'S069X7S'
'S062X1D'	'S06324S'	'S06358A'	'S064X1D'	'S06814S'	'S069X8A'
'S062X1S'	'S06325A'	'S06358D'	'S064X1S'	'S06815A'	'S069X8D'
'S062X2A'	'S06325D'	'S06358S'	'S064X2A'	'S06815D'	'S069X8S'
'S062X2D'	'S06325S'	'S06359A'	'S064X2D'	'S06815S'	'S069X9A'
'S062X2S'	'S06326A'	'S06359D'	'S064X2S'	'S06816A'	'S069X9D'
'S062X3A'	'S06326D'	'S06359S'	'S064X3A'	'S06816D'	'S069X9S'
'S062X3D'	'S06326S'	'S06360A'	'S064X3D'	'S06816S'	'S06A0XA'
'S062X3S'	'S06327A'	'S06360D'	'S064X3S'	'S06817A'	'S06A0XD'
'S062X4A'	'S06327D'	'S06360S'	'S064X4A'	'S06817D'	'S06A0XS'
'S062X4D'	'S06327S'	'S06361A'	'S064X4D'	'S06817S'	'S06A1XA'
'S062X4S'	'S06328A'	'S06361D'	'S064X4S'	'S06818A'	'S06A1XD'
'S062X5A'	'S06328D'	'S06361S'	'S064X5A'	'S06818D'	'S06A1XS'
'S062X5D'	'S06328S'	'S06362A'	'S064X5D'	'S06818S'	
'S062X5S'	'S06329A'	'S06362D'	'S064X5S'	'S06819A'	
'S062X6A'	'S06329D'	'S06362S'	'S064X6A'	'S06819D'	
'S062X6D'	'S06329S'	'S06363A'	'S064X6D'	'S06819S'	
'S062X6S'	'S06330A'	'S06363D'	'S064X6S'	'S06820A'	
'S062X7A'	'S06330D'	'S06363S'	'S064X7A'	'S06820D'	
'S062X7D'	'S06330S'	'S06364A'	'S064X7D'	'S06820S'	
'S062X7S'	'S06331A'	'S06364D'	'S064X7S'	'S06821A'	
'S062X8A'	'S06331D'	'S06364S'	'S064X8A'	'S06821D'	
'S062X8D'	'S06331S'	'S06365A'	'S064X8D'	'S06821S'	
'S062X8S'	'S06332A'	'S06365D'	'S064X8S'	'S06822A'	
'S062X9A'	'S06332D'	'S06365S'	'S064X9A'	'S06822D'	
'S062X9D’	'S06332S'	'S06366A'	'S064X9D'	'S06822S'	
'S062X9S'	'S06333A'	'S06366D'	'S064X9S'	'S06823A'	
'S06300A’	'S06333D'	'S06366S'	'S065X0A'	'S06823D'	
'S06300D'	'S06333S'	'S06367A'	'S065X0D'	'S06823S'	
'S06300S'	'S06334A'	'S06367D'	'S065X0S'	'S06824A'	
'S06301A'	'S06334D'	'S06367S'	'S065X1A'	'S06824D'	
'S06301D'	'S06334S'	'S06368A'	'S065X1D'	'S06824S'	
'S06301S'	'S06335A'	'S06368D'	'S065X1S'	'S06825A'	
'S06302A'	'S06335D'	'S06368S'	'S065X2A'	'S06825D'	
'S06302D'	'S06335S'	'S06369A'	'S065X2D'	'S06825S'	
'S06302S'	'S06336A'	'S06369D'	'S065X2S'	'S06826A'	

ICD-10-CM, International Classification of Diseases, Tenth Revision, Clinical Modification; TBI, traumatic brain injury.
