# Supplementary material for: Prevalence of Seizures in Hospitalizations with Traumatic Brain Injury: A U.S. Population-Based Study
Source: Neurotrauma Rep. 2025 Apr 9;6(1):291–7. doi: 10.1089/neur.2025.0001 (PMC12040531; doi:10.1089/neur.2025.0001)
Supplement: Supplementary Table S2 [file neur.2025.0001_supplementary_tables2.pdf]

Table S2. ICD-10-CM Codes Used for Diagnosis of Seizures or Epilepsy

'G40001'	'G40211'	'G4042'	'G40814'	'G40911'	'R5600'
'G40009'	'G40219'	'G40501'	'G40821'	'G40919'	'R5601'
'G40011'	'G40301'	'G40509'	'G40822'	'G40A01'	'R561'
'G40019'	'G40309'	'G40801'	'G40823'	'G40A09'	'R569'
'G40101'	'G40311'	'G40802'	'G40824'	'G40A11'	
'G40109'	'G40319'	'G40803'	'G40833'	'G40A19'	
'G40111'	'G40401'	'G40804'	'G40834'	'G40B01'	
'G40119'	'G40409'	'G40811'	'G4089'	'G40B09'	
'G40201'	'G40411'	'G40812'	'G40901'	'G40B11'	
'G40209'	'G40419'	'G40813'	'G40909'	'G40B19'	

ICD-10-CM, International Classification of Diseases, Tenth Revision, Clinical Modification.
